# Digital Mental Health: A Narrative Review of FDA-Authorized Products for Psychiatric Treatment and Diagnostic Support

**DOI:** 10.3390/brainsci16060576

**Published:** 2026-05-28

**Authors:** Jehad Albitar, Krista Ulisse, Christopher Feghali, Dorothy van Oppen, Matthew Zell, Daniel Elswick

**Affiliations:** Department of Behavioral Medicine and Psychiatry, Rockefeller Neuroscience Institute, West Virginia University, Morgantown, WV 26506, USA; jehad.albitar@hsc.wvu.edu (J.A.); cfeghal1@hsc.wvu.edu (C.F.); dorothy.vanoppen@hsc.wvu.edu (D.v.O.); mszell@hsc.wvu.edu (M.Z.); delswick@hsc.wvu.edu (D.E.)

**Keywords:** digital mental health, Digital Therapeutics (DTx), FDA-authorized digital therapeutics, prescription digital therapeutics, Software as a Medical Device (SaMD)

## Abstract

**Objective:** To identify FDA-authorized prescription or clinician-directed digital mental health products, including applications and software-enabled devices, for psychiatric treatment or diagnostic support. **Methods:** FDA Medical Device Databases were searched for records associated with nine product codes relevant to digital mental health. Eligible records were consolidated into distinct product families. Peer-reviewed literature and FDA documentation were reviewed to characterize indications, authorization pathways, and the quality of supporting evidence. **Results:** Sixteen digital mental health products and software-enabled devices authorized by the FDA met the inclusion criteria. Twelve products supported treatment for substance use disorders, insomnia, attention-deficit/hyperactivity disorder (ADHD), posttraumatic stress disorder (PTSD)-related conditions, major depressive disorder, generalized anxiety disorder, and postpartum depression. Four products provided diagnostic support for ADHD or autism spectrum disorder. Authorization was granted through either 510(k) clearance or De Novo classification, with considerable variation in the quantity, independence, and clinical relevance of supporting evidence. **Conclusions:** FDA marketing authorization does not uniformly indicate clinical effectiveness. Because 510(k) clearance, De Novo classification, and premarket approval (PMA) reflect distinct regulatory standards, clinicians should interpret digital mental health products in the context of the specific authorization pathway, product indication, and supporting evidence base.

## Simple Summary

Digital mental health products have the potential to address care gaps and barriers to mental health treatment by providing self-administered therapies and exercises. This narrative review identifies 16 FDA-authorized prescription or clinician-directed digital mental health products and software-enabled devices for psychiatric treatment or diagnostic support. Because the included products were authorized through both 510(k) clearance and De Novo classification pathways, “FDA-authorized” is used as the umbrella term, while “FDA-cleared” is reserved for 510(k) products. Treatment-supporting products addressed a range of clinical psychiatric conditions, including substance use disorders, major depressive disorder, generalized anxiety disorder, postpartum depression, insomnia, attention-deficit/hyperactivity disorder, and posttraumatic stress disorder. Products supporting diagnosis were identified for attention-deficit/hyperactivity disorder and autism spectrum disorder. While adoption is in its early stages, reimbursement pathways for digital therapeutics are emerging. The Centers for Medicare & Medicaid Services (CMS) has introduced new Medicare billing codes for digital mental health treatment devices furnished incident to behavioral health services, and Cigna Healthcare has introduced commercial coverage criteria for qualifying prescription digital therapeutics. These developments may reflect a trend toward broader adoption in the future. The review concludes that the supporting evidence base varies across these products. FDA 510(k) clearance does not necessarily require direct evidence of clinical effectiveness, because substantial equivalence may be sufficient in many cases. It is important to distinguish among FDA 510(k) clearance, FDA approval (PMA), and FDA grant of a De Novo classification request, as they represent fundamentally different regulatory standards. Further high-quality trials using final marketed products, appropriate comparators, longer follow-up, and clinically meaningful outcomes are needed for many products. Clinicians should interpret FDA marketing authorization as a regulatory permission to market, not as evidence of clinical effectiveness. The strength of the supporting evidence varies substantially across products and should be appraised independently of the authorization pathway.

## 1. Introduction

### 1.1. Access

Access to mental health treatment continues to present significant challenges, as office-based practices of psychiatrists and therapists are disproportionately concentrated in higher-income communities. Specifically, 25.3% of the wealthiest communities have psychiatrist practices, compared to only 8.0% of the poorest communities [1]. Despite the expansion of telemental health services, these access disparities persist. Recent evidence indicates that higher socioeconomic groups have disproportionately benefited from the expansion of telemental health services [2].

### 1.2. Terminology

The International Medical Device Regulators Forum and FDA define Software as a Medical Device as software intended for one or more medical purposes that performs those purposes independently of a hardware medical device [3,4]. This broad category includes digital therapeutics (DTx), which are evidence-based therapeutic interventions delivered via software to prevent, manage, or treat medical conditions [5]. Prescription digital therapeutics (PDTs), a subset of DTx, typically require a clinician’s prescription and, when making regulated medical-device claims, FDA marketing authorization.

### 1.3. FDA-Authorized Digital Mental Health Products

FDA-authorized digital mental health products encompass treatment-supporting products for substance use disorder, opioid use disorder, insomnia, attention-deficit/hyperactivity disorder (ADHD), depression, anxiety, and postpartum depression. Several platforms deliver cognitive behavioral therapy via software-based interventions [6]. Additionally, FDA-authorized digital mental health products include diagnostic-support products for autism spectrum disorder (ASD) and attention-deficit/hyperactivity disorder (ADHD).

### 1.4. Regulatory Pathways

The distinction between FDA clearance and FDA approval can be confusing for both healthcare providers and patients. It is essential to differentiate these regulatory pathways, as they are not interchangeable. Digital products may receive FDA marketing authorization through three primary pathways: the 510(k) premarket notification (yielding FDA clearance), the De Novo classification request (yielding FDA grant of classification), and premarket approval (PMA, yielding FDA approval), which is required for the highest-risk devices and necessitates “valid scientific evidence” of safety and effectiveness [5,6,7,8].

**Figure 1:**
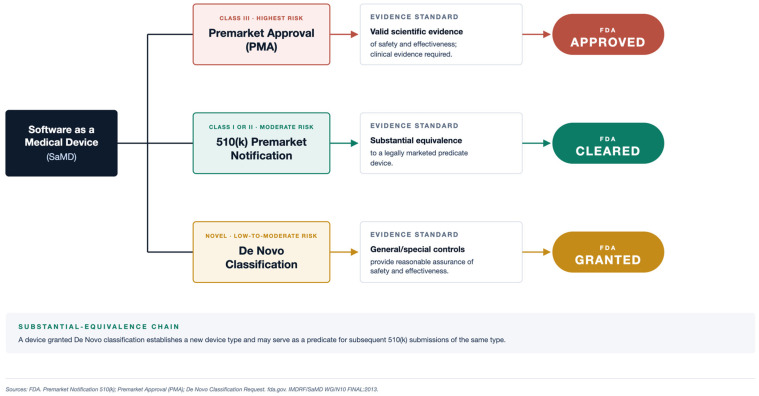
FDA regulatory pathways for Software as a Medical Device (SaMD) in digital mental health.

FDA approval is granted through the premarket approval pathway for high-risk Class III devices, whereas FDA clearance is provided via the 510(k) pathway based on substantial equivalence to a legally marketed predicate device [9]. In contrast to both pathways, the De Novo classification request applies to novel low- to moderate-risk devices for which no legally marketed predicate exists. A successful De Novo decision is described by the FDA as a ‘grant’ (21 CFR 860.260(a)), and the device is concurrently ‘classified’ into Class I or Class II [7,8]. Once granted, a De Novo device may serve as the predicate for subsequent 510(k) submissions, allowing subsequent devices to obtain 510(k) clearance through substantial-equivalence arguments rather than through a new De Novo review. Additional details are provided in Figure 1.

### 1.5. Review Objectives

This narrative review aims to identify FDA-authorized digital mental health products used for psychiatric treatment and diagnostic support, summarize their regulatory pathways and supporting evidence, and highlight limitations relevant to clinical interpretation.

## 2. Method

Design: narrative review.

Screening and data extraction:

FDA Medical Device Databases served as the sole source for product identification. Because these databases do not include a dedicated psychiatry or behavioral health panel for digital mental health products, relevant software and software-enabled devices are commonly indexed under the Neurology panel. Accordingly, records were retrieved from the FDA bulk Premarket Notification listing from January 1996 through 14 May 2026, filtered to the Neurology panel, and then refined using product-code filters corresponding to digital mental health-relevant device categories.

**Figure 2:**
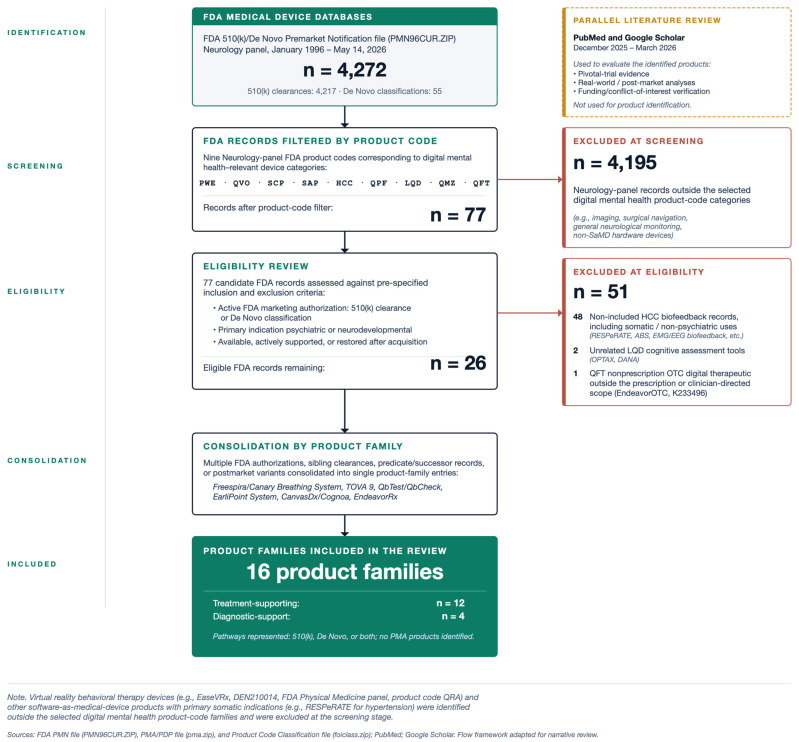
Study identification, screening, eligibility review, and product-family consolidation.

This initial Neurology-panel search yielded 4272 records, including 4217 510(k) clearances and 55 De Novo classifications. Records were then filtered to nine product codes: PWE, QVO, SCP, SAP, HCC, QPF, LQD, QMZ, and QFT. This yielded 77 candidate records. After eligibility review using prespecified inclusion and exclusion criteria, 51 records were excluded: 48 HCC biofeedback records addressing somatic or nonpsychiatric indications, 2 unrelated cognitive assessment tools under product code LQD, and 1 QFT record, EndeavorOTC (K233496, 2024), which was cleared for over-the-counter use and fell outside this review’s prescription or clinician-directed scope. The remaining 26 eligible records were consolidated into 16 unique FDA-authorized digital mental health products by merging multiple FDA authorizations, sibling clearances, or post-market variants belonging to the same product family, including Freespira, TOVA, QbTest, EarliPoint System, CanvasDx, and EndeavorRx. Virtual reality behavioral therapy devices, such as EaseVRx (DEN210014; FDA Physical Medicine panel; product code QRA) [10], and other software-as-medical-device products with primary somatic indications, such as RESPeRATE for hypertension [11], were outside the selected product-code categories and were excluded at the screening stage. HCC was retained in the initial screen because psychiatric biofeedback products could be embedded among broader biofeedback authorizations; nonpsychiatric HCC records were excluded during eligibility review. An adapted narrative-review flow diagram summarizing identification, screening, eligibility, consolidation, and inclusion is presented in Figure 2.

PubMed and Google Scholar were searched in parallel from December 2025 through March 2026, with supplementary verification through May 2026, to support the literature review and evidence appraisal for each identified product. Search terms included “digital mental health,” “digital therapeutics,” “prescription digital therapeutics,” “software as a medical device,” “SaMD,” and “mental health applications,” along with product-specific names identified through the FDA database queries. These searches were used to retrieve pivotal trials, real-world and post-market analyses, systematic reviews, and meta-analyses, and to verify authorship and conflict-of-interest disclosures. They were not used as primary sources for product identification. Non-peer-reviewed sources, including press releases, manufacturer webpages, payer policies, and news reports, were used only as contextual grey literature to verify regulatory status, commercial availability, acquisition history, reimbursement policy, or implementation context. These sources were not used as primary evidence for conclusions about clinical efficacy, diagnostic accuracy, or safety.

Product eligibility was assessed by the primary author and subsequently verified by coauthors. Discrepancies in product inclusion, regulatory pathway classification, or evidence interpretation were resolved through consensus review of FDA documentation and primary literature. No additional FDA product-identification records were incorporated after the 14 May 2026 FDA bulk-file cutoff.

**FDA product codes were defined as follows:** *PWE, computerized behavioral therapy device for substance use disorders; QVO, computerized behavioral therapy device for insomnia; SCP, computerized behavioral therapy device for generalized anxiety disorder; SAP, computerized behavioral therapy device for psychiatric disorders; HCC, biofeedback device; QPF, pediatric autism spectrum disorder diagnosis aid; LQD, computerized cognitive assessment aid; QMZ, vibrating tactile stimulator for nightmare disorder associated with PTSD; QFT, digital therapy device for ADHD.*

The inclusion criteria are as follows:

- Digital mental health products that have received FDA marketing authorization in the United States, including 510(k) clearance or De Novo classification, regardless of authorized age group, as identified in the FDA Medical Device Databases.
- Products were considered active if an official product or manufacturer website, or a current manufacturer/acquirer page, indicated ongoing public-facing availability or support at the time of review. Commercial availability was not independently verified through prescribing, payer, or procurement channels.
- Where a single product held more than one FDA marketing authorization (e.g., separate clearances for diagnostic and treatment-evaluation indications or successive 510(k) clearances of the same product family), these were consolidated and reported as a single entry.
- For products whose original developers underwent bankruptcy, acquisition, or corporate restructuring during the review period, active status was assessed based on the successor entity’s current commercial presence.

Exclusion criteria:

- Digital mental health products in the United States that have not received FDA marketing authorization.
- SaMD were excluded if their primary FDA-authorized indication was a somatic condition (e.g., chronic pain or hypertension), even where the mechanism of action involved behavioral or psychological components.
- Over-the-counter (non-prescription, consumer-facing) digital therapeutics were excluded, as the review focuses on prescription digital therapeutics and clinician-directed diagnostic-support products.

Evidence-appraisal approach:

Because this was a narrative review rather than a formal systematic review, we did not conduct GRADE, RoB 2, ROBINS-I, AMSTAR, or ROBIS assessments. Instead, we used a pragmatic narrative evidence-appraisal framework based on study design, comparator quality, sample size, endpoint interpretation, product-version relevance, independent replication, real-world generalizability, and sponsor involvement. Products were categorized as having moderate, limited-to-moderate, or limited evidence. Diagnostic-support products were appraised separately as diagnostic-support evidence because their clinical role differs from that of treatment products and should not be interpreted as a stand-alone diagnostic replacement.

AI-assisted tools:

AI-assisted tools were used to support literature organization, citation checking, data-extraction cross-checks, grammar editing, and simulated reviewer feedback. OpenEvidence (version generation 2.8.x) was used from December 2025 to March 2026 for data extraction, citation verification, and simulated peer-review feedback; DoximityGPT from January to February 2026 for the clinical literature identification and data extraction; Claude (Anthropic; Opus 4.6/4.7, March to May 2026) for data extraction, citation verification, figure formulation and simulated peer-review feedback; and Grammarly throughout drafting for grammar checking, paraphrasing, and editing. These tools were not used to determine product eligibility, make final evidence judgments, or generate unverified scientific claims. All AI-assisted outputs were independently verified against FDA documentation, peer-reviewed literature, or other primary sources before inclusion. No AI system met authorship criteria or is listed as an author.

## 3. Results

**Figure 3:**
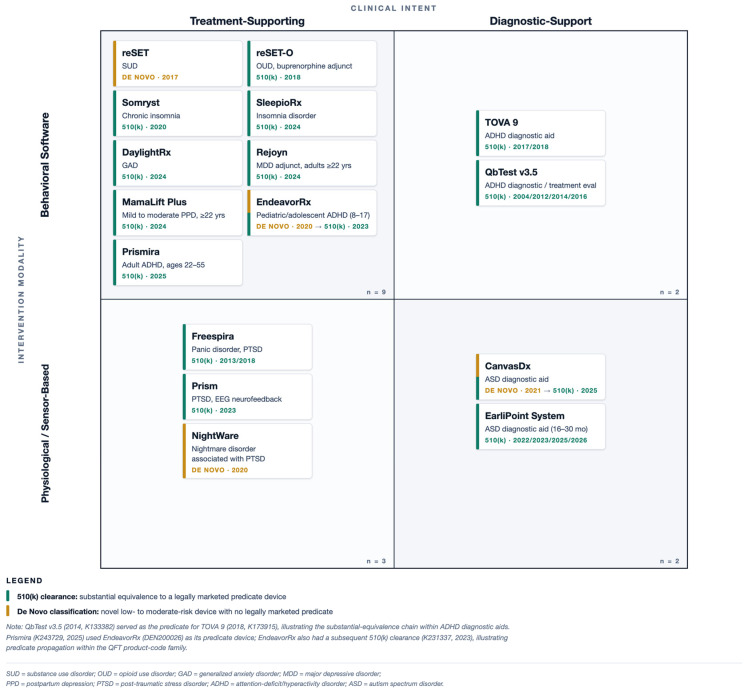
Study identification, screening, eligibility review, and product-family consolidation.

FDA-authorized digital mental health products, including applications and software-enabled devices, met the inclusion criteria. Treatment-supporting products addressed substance use disorders (*n* = 2), insomnia (*n* = 2), ADHD (*n* = 2), PTSD-related conditions (*n* = 3), major depressive disorder (*n* = 1), generalized anxiety disorder (*n* = 1), and postpartum depression (*n* = 1). Diagnostic-support products addressed ADHD (*n* = 2) and autism spectrum disorder (*n* = 2). Products were authorized through both 510(k) clearance and De Novo classification pathways, with multiple FDA records consolidated into product-family entries where appropriate. Active official product or manufacturer web presence was identified for all 16 included products at the time of review. This was interpreted only as evidence of current public-facing product or manufacturer presence, not as confirmation of payer coverage, prescribing availability, clinical adoption, or uninterrupted commercial access. Additional details are provided in Table 1 and Table 2 and Figure 3.

Mental health-related FDA-authorized digital mental health products can be broadly categorized as follows:

1. Computer-based cognitive behavioral therapy (CBT):
  ■ reSET: Substance use disorder (SUD) [12].
  ■ reSET-O: Opioid use disorder (OUD) [13].
  ■ Rejoyn: Major depressive disorder (MDD) [14].
  ■ DaylightRx: Generalized anxiety disorder (GAD) [15].
  ■ MamaLift Plus: Postpartum depression (PPD) [16].
  ■ Somryst: Insomnia [17].
  ■ SleepioRx: Insomnia [18].
2. Digital therapeutic software for ADHD:
  ■ EndeavorRx [19,20].
  ■ Prismira [21].
3. Physiological monitoring, biofeedback devices, and wearable-enabled products:
  ■ Freespira: Panic disorder, PTSD [22,23].
  ■ NightWare: PTSD-related nightmares [24].
  ■ Prism: Posttraumatic stress disorder (PTSD) [25].
4. Diagnostic support for ASD:
  ■ CanvasDx [26,27]
  ■ EarliPoint System [28,29,30,31]
5. Computerized attention assessment for ADHD diagnosis:
  ■ QbTest v3.5: ADHD diagnostic-support product [32,33,34,35]
  ■ Test of Variables of Attention (TOVA) 9: ADHD diagnostic-support product [36,37].

- EndeavorRx is reported using the consolidated FDA-authorized prescription indication for ages 8–17 years, reflecting the original De Novo grant for ages 8–12 (DEN200026, 2020) [19] and subsequent 510(k) clearance extending the indication to ages 13–17 (K231337, 2023) [20]. EndeavorOTC (K233496, 2024), an over-the-counter sibling product from the same applicant, was identified in the FDA candidate screen but excluded as a non-prescription consumer-facing variant outside the review’s prescription and clinician-directed scope.
- CanvasDx is reported as the consolidated Cognoa ASD diagnostic-support product family, comprising the original De Novo grant (DEN200069, 2021) [26] and subsequent 510(k) clearance (K243558, 2025) [27].
- EarliPoint System is reported as the consolidated EarliTec Diagnostics ASD diagnostic-support product family, comprising four 510(k) clearances (K213882, 2022; K230337, 2023; K243891, 2025; K253442, 2026) [28,29,30,31]. All four records are under FDA product code QPF and the same applicant.
- QbTest v3.5 is reported as the consolidated Qbtech ADHD attention-assessment product family, comprising the original QbTest 510(k) clearance K040894 (2004) [32], the QbTest v3.5 clearances K122149 (2012) [33] and K133382 (2014) [34], and the at-home QbCheck sibling clearance K143468 (2016) [35]. All four records are under FDA product code LQD with the same applicant, Qbtech AB. Per the K143468 510(k) clearance letter [35], QbCheck is designated for prescription use under 21 CFR 801 Subpart D, clinician-ordered and clinician-interpreted, distinguishing it from nonprescription consumer products excluded from this review.

## 4. Evidence Review

### 4.1. Therapeutic Products:

#### 4.1.1. reSET

- Overview:
  - FDA authorization: De Novo, DEN160018 (2017) [12].
  - Use: Substance use disorder (SUD), excluding patients receiving opioid replacement therapy, individuals with alcohol as their sole substance, and those whose primary substance was opioids [38].
  - Treatment module: This product uses an integrated cognitive-behavioral therapy (CBT) based on the Community Reinforcement Approach (CRA), a contingency management (CM) system that offers motivational incentives to reinforce the mastery of concepts [38].
  - Structure and duration: The intervention spans 12 weeks and includes 61 therapy lessons that address cognitive-behavioral strategies, relapse prevention, behavioral risk reduction education, and psychosocial functioning. Contingency management is implemented, with rewards provided for lesson completion and negative urine drug screens [38].
- Evidence Quality [38]:
  - Study type: Real-world observational study.
  - Sample size: 602.
  - Control group: None.
- Evidence:
  - Real-world study of 602 patients; 52% completed all core modules; 74% retention at weeks 9–12; 62% abstinent at end of treatment [38].
  - A 50% reduction in hospital encounters at 6 months post-initiation; $3591 per-patient cost reduction over 6 months [39].
- Limitations:
  - The study is limited by a high dropout rate (48% non-completion), a real-world observational design without a control group, a heterogeneous population, absence of data on patients receiving alternative treatments, and reliance on self-reported outcomes. These factors collectively limit the generalizability of the findings [38].
  - Pear Therapeutics, the original developer of reSET and reSET-O, filed for Chapter 11 bankruptcy in April 2023 [40], leading to an interruption in product availability. PursueCare subsequently acquired these assets in December 2023 [41] and resumed patient access in August 2024 [42].

#### 4.1.2. reSET-O

- Overview:
  - FDA authorization: 510(k), K173681 (2018) [13].
  - Use: Opioid use disorder (OUD) as an adjunct to outpatient treatment that includes buprenorphine [43].
  - Treatment module: Similar to reSET, this product integrates cognitive-behavioral therapy (CBT) based on the Community Reinforcement Approach (CRA), a contingency management (CM) system that offers motivational incentives to reinforce the mastery of concepts [43].
  - Structure and duration: 12 weeks [43].
- Evidence Quality [43]:
  - Study type: Real-world observational study.
  - Sample size: 3144.
  - Control group: None.
- Evidence:
  - Real-world study of 3144 patients [43] found that 49% completed all modules, while 80% completed at least 8 of 67 therapeutic modules.
  - Abstinence during the final four weeks was 66% using the conservative ‘missing data positive’ imputation and 91% using the ‘missing data excluded’ method; participants meeting the responder definition (≥80% negative self-report or UDS) also reached 91% [43]. At 12 months, 28% fewer inpatient stays and 56% fewer hospital readmissions were observed, with a $2791 per-patient cost reduction compared to controls [43,44].
- Limitations:
  - High dropout rate (51% non-completion), absence of comparison groups [43].
  - The primary real-world evidence studies [43,44] were funded by Pear Therapeutics, Inc., the developer of reSET-O. All authors of [43] were Pear Therapeutics employees or contractors, and the lead authors of [43,44] were Pear Therapeutics employees, representing a potential industry conflict of interest.
  - As with reSET, Pear Therapeutics filed for Chapter 11 bankruptcy in April 2023 [40]. PursueCare subsequently acquired reSET-O and resumed patient access in August 2024 [41,42].

#### 4.1.3. Somryst

- Overview:
  - FDA authorization: 510(k), K191716 (2020) [17].
  - Use: Chronic insomnia [45].
  - Duration: 9 weeks [45].
  - Treatment module: Digital CBT-I-based product with sleep restriction, stimulus control, and cognitive restructuring principles [45].
- Evidence Quality:
  - Study type: Network meta-analysis [45] incorporating RCTs of Sleep Healthy Using the Internet (SHUTi) [46], an early web-based version of Somryst with equivalent content.
  - Sample size: Multiple trials pooled.
  - Control group: Varies across included trials; comparators included face-to-face CBT-I, pharmacotherapy, and waitlist control [45].
- Evidence:
  - Network meta-analysis found it had the highest probability of being the most effective treatment for insomnia compared to face-to-face CBT-I and FDA-approved sleep medications [45].
- Limitations:
  - Heterogeneity in control conditions and study designs; restricted to patients with primary or chronic insomnia, excluding insomnia comorbid with another condition; short-term outcomes excluded from data analysis; reliance on patient-reported sleep measures [45].
  - This network meta-analysis [45] was funded by Pear Therapeutics, Inc., the developer of Somryst, and three of six authors were Pear Therapeutics employees, representing a potential industry conflict of interest.
  - Pear Therapeutics, the developer of Somryst, filed for Chapter 11 bankruptcy in April 2023 [40]. Subsequently, Nox Health Group acquired the Somryst application [47].

#### 4.1.4. SleepioRx

- Overview:
  - FDA authorization: 510(k), K233577 (2024) [18].
  - Use: Insomnia [18].
  - Module: Digital CBT-I-based product. Interventions are self-administered through audio, visual, and interactive modalities, including [48] the following:
    - Cognitive techniques: These include cognitive restructuring and paradoxical intention.
    - Behavioral techniques: These include stimulus control, sleep restriction, and sleep hygiene.
    - Physiological technique: This category includes progressive muscle relaxation.
  - Duration: 90 days [18].
- Evidence Quality:
  - Study type: Two RCTs [48,49]
  - Sample size: *n* = 164 [49]; *n* = 336 [48].
  - Control group: Credible psychological placebo (imagery relief therapy, IRT) + TAU [49]; sleep hygiene education [48].
- Evidence:
  - A total of 76% of patients achieved healthy sleep (SE > 80%) compared with 29% in the imagery relief therapy placebo group and 18% in the usual care group [49].
  - A decentralized nationwide randomized controlled trial demonstrated that SleepioRx led to significant and sustained improvements in insomnia severity compared to sleep hygiene education. Participants receiving SleepioRx were 2.5 times more likely to respond and 5.8 times more likely to achieve remission at week 10, with these outcomes maintained through the 24-week follow-up [48].
- Limitations:
  - The evidence base primarily originates from efficacy trials conducted with selected populations. Several of these trials utilized prototype versions on digital platforms that differ from the final FDA-authorized version [48].
  - The pivotal decentralized randomized controlled trial (RCT) employed sleep hygiene education as the comparator, rather than face-to-face cognitive behavioral therapy for insomnia (CBT-I) or pharmacotherapy. This choice restricts direct head-to-head comparisons with established gold-standard treatments.
  - The foundational trial [49] was not industry-funded; however, two authors held leadership roles at Sleepio Limited, the software developer. The pivotal trial [48] involved multiple authors with financial relationships with Big Health, Inc., the developer of SleepioRx, including employment, equity ownership, stock options, grant funding, and consulting arrangements. These relationships represent potential conflicts of interest.

#### 4.1.5. EndeavorRx

- Overview:
  - FDA authorization: De Novo, DEN200026 (2020) [19], and 510(k), K231337 (2023) [20].
  - Use: ADHD in pediatric and adolescent (ages 8–17) [20].
  - Duration [19]:
  - Initial: 4-week treatment, 25 min/day, 5 days/week.
  - Pause: 4-week break after initial 4 weeks.
  - Repeat: Optional second 4-week course if needed.
  - Treatment module: Game-based digital attention training methods through engaging game mechanics [19].
- Evidence Quality:
  - Study type: Systematic review and meta-analysis [50].
  - Sample size: 14 RCTs pooled; *n* = 1183 total participants [50].
  - Control group: Sham/active controls included in RCTs [50].
- Evidence:
  - A systematic review and meta-analysis of RCTs of game-based digital therapeutics, including EndeavorRx, showed improvement in inattention and hyperactivity/impulsivity versus controls as assessed by parents and teachers; however, medication remained more effective [50].
- Limitations:
  - Limited real-world transfer [51].

#### 4.1.6. Prismira

- Overview:
  - FDA authorization: 510(k), K243729 (2025) [21].
  - Use: ADHD in adults aged 22–55 years with primarily inattentive or combined-type ADHD; indicated to improve attention function. The FDA summary notes that patients may not display benefits in typical behavioral symptoms, such as hyperactivity [21].
  - Duration: 9 weeks [21].
  - Treatment module: The intervention consists of a mobile software-as-medical-device in which patients complete an ordered sequence of brief touchscreen games, each lasting 1–5 min, with visual and optional auditory feedback, for a total of approximately 15 min per day over 9 weeks [21].
- Evidence Quality:
  - Study type: GAMES Study, a randomized, double-blind, parallel-group, multicenter, sham-controlled pivotal trial [21].
  - Sample size: 560 adults; 456 completed treatment and Day 63 follow-up and were included in the ITT population, including 224 active and 232 sham-control participants [21].
  - Control group: Sham digital program [21].
- Evidence:
  - The primary endpoint was met. The TOVA-ACS (TOVA-Attention Comparison Score) improved significantly at Day 63 in the active group relative to the sham group (least-squares mean improvement of 1.09 versus 0.30; least-squares mean difference of 0.78, with a 95% CI 0.15–1.41; *p* = 0.0149). Additionally, sensitivity analysis that included outliers also yielded significant results (*p* = 0.0452) [21].
  - The active-arm improvement did not reach the recognized TOVA-ACS minimal clinically important difference of 1.4 points, although it exceeded the improvement reported for the predicate device, EndeavorRx (0.93-point change) [21]. Most clinician- and patient-reported secondary endpoints favored active treatment but were not statistically significant; blinded clinician CGI-I was significantly better in the active arm (*p* = 0.0087) [21].
  - No serious adverse events were reported. Two treatment-related adverse events occurred in the active-therapy safety population: both were reports of decreased frustration tolerance during gameplay that resolved at the conclusion of gameplay; treatment compliance averaged 97.2% of expected minutes [21].
- Limitations:
  - Pivotal-trial results are currently available through the FDA 510(k) decision summary and manufacturer-reported data; no peer-reviewed full publication of the GAMES Study was identified at the time of synthesis.
  - The primary endpoint was a digitally administered attention task, and the active-arm improvement did not reach the established MCID for that instrument.
  - Statistically significant between-group differences were not observed for most clinician- and patient-reported secondary endpoints.
  - The evidence may not generalize outside adults aged 22–55 years with inattentive or combined-type ADHD, and the device is not intended for users with photosensitive epilepsy, color blindness, or physical limitations affecting mobile-device use.
  - The pivotal trial was sponsored and conducted by Lumos Labs Medical, and the FDA summary is currently the only available source.

#### 4.1.7. DaylightRx

- Overview:
  - FDA authorization: 510(k), K233872 (2024) [15].
  - Use: Generalized Anxiety Disorder [6].
  - Duration: 90 days [15].
  - Treatment module [52]:
  - Digital CBT-based product comprising four modules on applied relaxation, stimulus control, cognitive restructuring, and imaginal exposure, each approximately 10 to 20 min in duration.
- Evidence Quality [52]:
  - Study type: RCT.
  - Sample size: 256.
  - Control group: Waitlist control.
- Evidence:
  - At post-intervention (week 6), 61% of digital CBT participants achieved remission versus 31% of waitlist controls (OR 3.99; *p* < 0.001); at follow-up (week 10), this increased to 71% versus 33% [52].
- Limitations:
  - Self-reported symptoms, the study utilized a waitlist control group (does not account for expectancy or attention effects) [52].
  - Notably, this trial was published in 2020 [52], predating FDA clearance of DaylightRx in 2024, and may reflect a prototype version delivered on a different platform than the final authorized product, a limitation recognized in the broader digital therapeutics literature.
  - The primary evidence source [52] involved multiple authors with financial relationships with Big Health, Inc., the developer of DaylightRx, including employment, equity ownership, and a co-founder role, representing a potential industry conflict of interest.

#### 4.1.8. Rejoyn

- Overview:
  - FDA authorization: 510(k), K231209 (2024) [14].
  - Use: Adjunctive treatment for major depressive disorder (MDD) in adults aged ≥ 22 years who are receiving antidepressant medication [14,53].
  - Duration: 6 weeks, with 4 weeks extension period [53].
  - Treatment module [53]:
    - CBT-based lessons: Deliver core cognitive-behavioral therapy content aimed at alleviating depressive symptoms.
    - Emotional faces memory training (EFMT): Designed to improve cognitive control over emotional information processing, which potentially promotes brain connectivity and neuroplasticity.
- Clinical rationale:
  - The intervention is hypothesized to target emotional information processing and may influence amygdala–prefrontal circuitry relevant to emotion regulation [53,54].
- Evidence Quality [53]:
  - Study type: Phase 3 RCT.
  - Sample size: 386.
  - Control group: Sham digital therapeutic.
- Evidence:
  - In Phase 3’s randomized controlled trial (the Mirai study; N = 386 ITT, *n* = 354 mITT), the primary mITT analysis showed a MADRS reduction of 9.03 points with Rejoyn versus 7.25 points with sham (difference −1.78; *p* = 0.057), which did not reach statistical significance. The supportive ITT analysis favored Rejoyn with a between-group difference of −2.12 points (*p* = 0.021) [53].
- Limitations:
  - The primary mITT endpoint did not reach statistical significance (*p* = 0.057) [53], and limited long-term effectiveness data are available.
  - The primary evidence source [53] was funded by Otsuka Pharmaceutical Development & Commercialization, Inc. and Click Therapeutics, Inc., the co-developers of Rejoyn. The majority of authors were employees of these companies, and remaining authors held consulting relationships with the developers, representing a potential industry conflict of interest.

#### 4.1.9. MamaLift Plus

- Overview:
  - FDA authorization: 510(k), K223515 (2024) [16].
  - Use: Mild to moderate postpartum depression in adults aged ≥ 22 years [16,55].
  - Duration: 8 weeks [55].
  - Treatment module [55]:
    - CBT as the foundational component, augmented by behavioral activation therapy (BAT), interpersonal therapy (IPT), and dialectical behavior therapy (DBT) elements.
    - Digital CBT-based application comprises one module per week. Booster sessions are available on an optional basis to reinforce acquired skills.
- Evidence Quality: [55]
  - Study type: Double-blind Phase 3 pivotal RCT.
  - Sample size: 141.
  - Control group: Sham digital app (no CBT content).
- Evidence:
  - A total of 86.3% of MamaLift Plus participants achieved a clinically meaningful improvement (≥4-point EPDS reduction) compared with 23.9% in the sham control arm [55].
- Limitations:
  - The trial was limited to participants with mild to moderate postpartum depression, which may limit generalizability [55].
  - No long-term follow-up data beyond 8 weeks; decentralized recruitment via social media may limit generalizability to real-world clinical settings. [55].
  - The primary evidence source was funded by Curio Digital Therapeutics, the developer of MamaLift Plus, and all authors were employees with equity or stock options in the company, representing a potential industry conflict of interest [55].

#### 4.1.10. Freespira

- Overview:
  - FDA authorization: 510(k) clearance for panic disorder in 2013, K131586 [22], with PTSD added through a separate 510(k) clearance in 2018, K180173 [23].
  - Use: Panic disorder, PTSD [56].
  - Duration: 28 days [56].
  - Treatment module [56]:
    - Uses capnometry-guided respiratory intervention, providing real-time feedback on respiratory rate and exhaled CO2 levels, aiming to normalize breathing patterns and assist patients in better managing symptoms associated with stress, anxiety, and panic.
    - Each session is 17 min; two sessions are recommended daily.
- Evidence Quality: [56]
  - Study type: Real-world effectiveness study.
  - Sample size: 1569.
  - Control group: None.
- Evidence:
  - Panic disorder: 50.2% reduction in total Panic Disorder Severity Scale (PDSS) scores, with 65.3% achieving a treatment response [56].
  - PTSD: 41.1% reduction in total PTSD Checklist for DSM-5 scores, with a 72.4% treatment response rate [56].
- Limitations:
  - Non-randomized design; no active control or respiratory placebo group, no extended follow-up to assess durability of effects [56]. The lack of a respiratory placebo control is a significant limitation, as breathing exercises alone may produce benefits independent of the biofeedback mechanism [56].
  - The primary evidence source [56] was funded by Freespira, Inc., the developer of the intervention. Three of four authors were Freespira employees with salary and equity, and the remaining author was a paid scientific advisor with stock options, representing a potential industry conflict of interest.

#### 4.1.11. NightWare

- Overview:
  - FDA authorization: De Novo, DEN200033 (2020) [24].
  - Use: Nightmare disorder associated with PTSD [57].
  - Module: AI-driven Apple Watch system monitors heart rate and motion during sleep; delivers calibrated vibrotactile stimulation when a nightmare-associated “Stress Index” threshold is crossed, interrupts nightmares without waking the patient [57].
- Evidence Quality [57]:
  - Study type: Randomized double-masked sham-controlled trial.
  - Sample size: 65 veterans with impaired sleep secondary to trauma-related nightmares.
  - Control group: Sham wearable device.
- Evidence: [57]
  - Randomized, double-masked, sham-controlled trial; *n* = 65 veterans (30 active, 35 sham) with PTSD and frequent trauma-related nightmares (>7/month); 30-day trial duration. Both active and sham conditions demonstrated statistically significant within-person improvement across sleep quality, PTSD symptoms, and quality of life measures from baseline.
  - In the full sample (intent-to-treat), the active condition showed a consistently greater magnitude of improvement than sham across all measures; however, no between-group difference reached statistical significance, including the primary outcome.
- Limitations:
  - The primary ITT outcome did not reach statistical significance; between-group effects were observed only in a post hoc high-usage subgroup, which limits causal interpretation and generalizability [57]. Therefore, the De Novo authorization should be interpreted as FDA marketing authorization under a benefit-risk assessment, rather than as a confirmation of conclusive clinical effectiveness.
  - No objective sleep data (e.g., polysomnography or actigraphy) were collected; the study cannot confirm whether the device directly interrupted nightmare-associated sleep stages or confirm the physiological basis of the “Stress Index” algorithm [57]. Findings are specific to a predominantly VA veteran population with PTSD; generalizability to civilian populations or nightmare disorder without PTSD is unknown [57].
  - The NightWare Likert (NWL) scale used as a secondary outcome has not been externally validated [57].
  - Small sample size, *n* = 65 completers. No long-term follow-up; the durability of the benefit beyond 30 days is not established [57].
  - The primary evidence source was funded by NightWare, Inc., the device’s developer. However, neither author received direct compensation from the company, and NightWare did not participate in data analysis or reporting, representing a reduced but nonetheless present potential industry conflict of interest [57].

#### 4.1.12. Prism

- Overview:
  - FDA authorization: 510(k), K222101(2023) [25].
  - Use: Adjunctive neurofeedback treatment for posttraumatic stress disorder (PTSD), used in addition to standard of care [58].
  - Duration: 8 weeks [58].
  - Treatment module:
    - Software-based neurofeedback device running on a standard computer with supported EEG hardware; 15 sessions of approximately 25 min delivered twice weekly on non-consecutive days over 8 weeks, with optional booster sessions.
    - Delivers operant-conditioning neurofeedback based on amygdala-derived electrical fingerprint (EFP) signals, with real-time visual and auditory feedback responsive to the patient’s amygdala-derived EFP activity [58].
- Evidence Quality:
  - Study type: Prospective, single-arm, multisite, multinational, open-label trial [58].
  - Sample size: 79 enrolled (full analysis set); 66 in the effectiveness analysis set [58].
  - Control group: None [58].
- Evidence:
  - Primary endpoint met: 66.7% of participants in the effectiveness analysis set achieved a clinically meaningful CAPS-5 response (Clinician-Administered PTSD Scale for DSM-5) at 3-month follow-up [58].
  - Mean CAPS-5 score decreased by 13.5 points at the 3-month follow-up, more than twice the predefined minimally clinically important difference; CAPS-5, PCL-5 (PTSD Checklist for DSM-5), and PHQ-9 (Patient Health Questionnaire-9) scores showed statistically significant improvement from baseline to 8 weeks and 3-month follow-up; and the CAPS-5 remission rate was 31.8% at the 3-month follow-up [58].
- Limitations:
  - Single-arm, open-label design, which limits causal interpretation because there was no placebo, sham neurofeedback, or active control arm [58].
  - The study evaluated Prism as an adjunct to standard of care, making it difficult to isolate the independent effect of neurofeedback from concurrent PTSD treatment [58]. Indicated only for chronic PTSD; not validated for acute PTSD [58].
  - A statistically significant difference in response was observed between US and OUS sites at the 3-month follow-up (*p* = 0.0211), but not at 8 weeks (*p* = 0.5494); four of five sites were located in Israel. When stratified by trauma type, the difference was not present for non-military trauma. However, this may still limit the generalizability of the findings to US clinical practice [58].
  - Multiple authors [58] disclosed financial relationships with the device developer/manufacturer, including reimbursement consulting, compensation for statistical analysis, and research support, representing a potential industry-related conflict of interest.

### 4.2. Diagnostic-Support Products:

#### 4.2.1. CanvasDx

- Overview:
  - FDA authorization: De Novo, DEN200069 (2021) [26], and 510(k), K243558 (2025) [27].
  - Use: ASD diagnosis aid [59].
  - Module:
    - AI-based behavioral analysis software employs three distinct inputs: a caregiver questionnaire, two brief home videos of the child, and a healthcare provider questionnaire. These inputs are analyzed utilizing a gradient-boosted decision tree machine learning algorithm to generate one of three outcomes: ASD positive, ASD negative, or indeterminate [59].
  - Duration: Asynchronous; caregiver questionnaire (~5 min) and two brief home videos submitted via app; clinician questionnaire (~10 min) completed via web portal [60].
- Evidence Quality:
  - Study type: Pivotal diagnostic accuracy study [59]; real-world post-market analysis [60].
  - Sample size: 425 [59]; 254 [60].
  - Control group: None, as the reference standard is a specialist diagnosis.
- Evidence:
  - In a study of 425 children (29% ASD), the software had a positive predictive value of 80.8% and a negative predictive value of 98.3% [59].
  - Real-world post-market data demonstrated improved performance, with an NPV of 97.6%, PPV of 92.4%, and 63.0% of cases receiving determinate results [60].
- Limitations:
  - Indeterminate outputs occur in a substantial proportion of cases, especially when presentations are complex or comorbidities exist. Because positive predictive value depends on the underlying prevalence of ASD in the tested population, the PPV reported in the pivotal study should be interpreted in the context of that study’s enriched clinical sample and may not generalize to lower-prevalence screening settings [59,60].

#### 4.2.2. EarliPoint System

- Overview:
  - FDA authorization: 510(k), K213882 (2022) [28], K230337 (2023) [29], K243891 (2025) [30], and K253442 (2026) [31].
  - Use: ASD diagnosis aid [61].
  - Module:
    - The system quantifies children’s visual focus on social stimuli by tracking eye movements while they watch brief video clips of naturalistic social scenes.
  - Duration: 14 video scenes (mean ~54 s each; total ~12 min) [61].
- Evidence Quality:
  - Study type: Multisite prospective double-blind Phase 3 diagnostic accuracy study [61].
  - Sample size: 475, aged 16–30 months.
  - Control group: None, as the reference standard is a specialist diagnosis.
- Evidence [61]:
  - Overall sensitivity was 71.0% and specificity was 80.7%. In the high-certainty subgroup of 335 children, sensitivity was 78.0% and specificity was 85.4%
  - Rationale is based on research demonstrating that reduced attention to eyes and faces during social interaction is a quantifiable early marker of ASD.
- Limitations:
  - This study’s limitation was that it was not designed for general population screening.
  - The lead and senior authors of [61] are scientific co-founders and equity holders of the company that developed the index device under review, serve as paid scientific consultants to that company, and hold multiple patents licensed to it. Two additional authors disclosed paid consultancy or institutional funding and stock from the same company. These relationships constitute a significant potential industry conflict of interest.

#### 4.2.3. Test of Variables of Attention (TOVA) 9

- Overview:
  - FDA authorization: Received 510(k) clearance (K170082) in 2017 [36]. A subsequent 510(k) for the unversioned T.O.V.A. product line (K173915) was granted in 2018 [37].
  - Use: ADHD diagnosis aid [36].
  - Duration: Approximately 21.6 min [36].
  - Module:
    - Presents visual or auditory stimuli in a Continuous Performance Test (CPT) paradigm, measuring response times, variability in response times, omission errors (indicative of inattention), and commission errors (indicative of impulsivity) [37].
- Evidence Quality:
  - Study type: Systematic review and meta-analysis [62].
  - Sample size: 19 studies; up to 819 cases and 835 controls pooled for sensitivity/specificity analyses [62].
  - Control group: Varies across included studies [62].
- Evidence:
  - Pooled sensitivity of 0.75 and specificity of 0.71 for the total/ADHD score across commercially available CPTs [62].
- Limitations:
  - Limited diagnostic utility as a stand-alone tool [62].
  - The primary evidence source [62] reported no funding for this work. Additionally, 6 of 12 authors reported no biomedical financial interests or potential conflicts of interest. Other authors disclosed pharmaceutical-industry relationships and publisher royalties; however, the disclosure did not specifically identify financial relationships with developers of CPT diagnostic tools.

#### 4.2.4. QbTest v3.5

- Overview:
  - FDA authorization: 510(k), K040894 in 2004 (original QbTest clearance) [32], K122149 in 2012 (for ADHD assessment/diagnosis) [33], K133382 in 2014 (for treatment evaluation) [34], and K143468 in 2016 (QbCheck at-home prescription sibling) [35].
    - Notably, K133382 (QbTest, 2014) [34] served as the predicate device for TOVA 9’s 510(k) clearance (K173915), demonstrating the substantial equivalence chain within the same device category. This regulatory pathway does not mandate independent clinical evidence of effectiveness for the authorized device.
  - Use: ADHD diagnosis aid; treatment evaluation [33,34].
  - Module:
    - Duration: Approximately 15–20 min [63].
    - Combines continuous performance test (CPT) with infrared motion tracking technology to measure the three cardinal symptoms: inattention, hyperactivity, and impulsivity [63,64].
- Evidence Quality:
  - Study type: Systematic review and meta-analysis [64].
  - Sample size: 15 studies [64].
  - Control group: Varies across included studies [64].
- Evidence:
  - A systematic review found an acceptable sensitivity (0.78) and specificity (0.70), indicating a moderate discriminative capacity [64].
- Limitations:
  - A systematic review of QbTest in children and adolescents with ADHD revealed limited diagnostic utility as a stand-alone tool [64].
  - The primary evidence source [64] was not commercially funded; however, some authors held prior professional relationships with the QbTest developer or its regulatory evaluation, representing a potential but limited conflict of interest.

**Table 2:** Evidence Summary and Narrative Evidence Appraisal of FDA-Authorized Digital Mental Health Products.

Product	FDA Auth.	Use	Mechanism/Module	Duration	Study Type/Sample	Key Evidence	Key Limitations	Evidence Appraisal
Computer-Based Cognitive Behavioral Therapy (CBT) (n = 7)								
reSET	De Novo, DEN160018 (2017) [12]	Substance use disorder (SUD), excluding patients on opioid replacement therapy, alcohol-only use, or primary opioid use [38]	CBT based on the Community Reinforcement Approach (CRA); a contingency management (CM) system that offers motivational incentives to reinforce the mastery of concepts [38]. 12 weeks; 61 therapy lessons addressing CBT strategies, relapse prevention, behavioral risk reduction, and psychosocial functioning [38].	12 weeks [38]	Real-world observational study [38]. n = 602. Control group: None.	52% completed all core modules; 74% retention at weeks 9–12; 62% abstinent at end of treatment [38]. 50% reduction in hospital encounters at 6 months post-initiation; $3591 per-patient cost reduction over 6 months [39].	High dropout rate (48% non-completion), real-world observational design without a control group, heterogeneous population, absence of data on patients receiving alternative treatments, reliance on self-reported outcomes [38].	Limited evidence [38,39]
reSET-O	510(k), K173681 (2018) [13]	Opioid Use Disorder (OUD), adjunct to outpatient treatment including buprenorphine [43]	CBT based on the Community Reinforcement Approach (CRA); a contingency management (CM) system that offers motivational incentives to reinforce the mastery of concepts [43].	12 weeks [43]	Real-world observational study [43]. n = 3144. Control group: None.	49% completed all modules; 80% completed at least 8 of 67 therapeutic modules. Abstinence at the final 4 weeks: 66% (conservative) and 91% (missing excluded); 28% fewer inpatient stays and 56% fewer hospital readmissions at 12 months; $2791 per-patient cost reduction compared to controls [43,44].	High dropout rate (51% non-completion), absence of comparison groups [43].	Limited evidence [43,44]
Somryst	510(k), K191716 (2020) [17]	Chronic Insomnia [45]	Digital CBT-I-based product with sleep restriction, stimulus control, and cognitive restructuring principles [45].	9 weeks [45]	Network meta-analysis [45] incorporating RCTs of SHUTi [46]. Sample: Multiple trials pooled. Comparators: face-to-face CBT-I, pharmacotherapy, waitlist control.	Highest probability of being the most effective treatment for insomnia compared to face-to-face CBT-I and FDA-approved sleep medications [45].	Heterogeneity in control conditions and study designs, focusing on primary or chronic insomnia populations, excluding comorbid insomnia and short-term outcomes (6–12 weeks), which limits comparability and generalizability; reliance on patient-reported sleep measures [45].	Moderate evidence [45,46]
SleepioRx	510(k), K233577 (2024) [18]	Insomnia [18]	Digital CBT-I-based product. Interventions are self-administered through audio, visual, and interactive modalities, including cognitive restructuring, paradoxical intention, stimulus control, sleep restriction, sleep hygiene, and progressive muscle relaxation [48].	90 days [18]	Two RCTs [48,49]; n = 164 [49]; n = 336 [48] Control: IRT + TAU [49]; sleep hygiene education [48].	76% vs. 29% (IRT placebo) vs. 18% (usual care) achieved SE > 80% [49]. 2.5 times more likely to respond and 5.8 times more likely to achieve remission at week 10; maintained through 24-week follow-up [48].	Evidence is based primarily on efficacy trials using prototype versions differing from the final FDA-authorized product [48]. Pivotal RCT used sleep hygiene education as a comparator rather than face-to-face CBT-I or pharmacotherapy [48].	Moderate evidence [48,49]
DaylightRx	510(k), K233872 (2024) [15]	Generalized Anxiety Disorder (GAD) [15]	Digital CBT-based product comprising four modules on applied relaxation, stimulus control, cognitive restructuring, and imaginal exposure, each approximately 10 to 20 min in duration [52].	90 days [15]	RCT [52]. n = 256. Control group: Waitlist control.	61% of digital CBT participants achieved remission versus 31% of waitlist controls (OR 3.99; *p* < 0.001) at week 6; increased to 71% versus 33% at week 10 [52].	Self-reported symptoms; waitlist control does not account for expectancy or attention effects [52]. Trial published in 2020, predating FDA clearance of DaylightRx in 2024, and may reflect a prototype version [52].	Moderate evidence [52]
Rejoyn	510(k), K231209 (2024) [14]	MDD, adjunct to antidepressant medication in adults aged ≥ 22 years [53]	CBT-based lessons to alleviate depressive symptoms; Emotional Faces Memory Training (EFMT) designed to improve cognitive control over emotional information processing, promoting brain connectivity and neuroplasticity [53].	6 weeks, with a 4-week extension period [53]	Phase 3 RCT [53]. N = 386 ITT; n = 354 mITT. Control group: Sham digital therapeutic [53]	mITT analysis: MADRS reduction of 9.03 points with Rejoyn versus 7.25 points with sham (difference −1.78; *p* = 0.057); did not reach statistical significance. Supportive ITT analysis: between-group difference of −2.12 points (*p* = 0.021) [53].	Primary mITT endpoint did not reach statistical significance (*p* = 0.057) [53]; limited long-term effectiveness data.	Limited-to-moderate evidence [53,54]
MamaLift Plus	510(k), K223515 (2024) [16]	Mild to moderate postpartum depression (PPD) [55]	CBT as the foundational component, augmented by behavioral activation therapy (BAT), interpersonal therapy (IPT), and dialectical behavior therapy (DBT) elements; one module per week; optional booster sessions [55].	8 weeks [55]	Double-blind Phase 3 pivotal RCT [55]. n = 141. Control group: Sham digital app (no CBT content).	86.3% of MamaLift Plus participants achieved a clinically meaningful improvement (≥4-point EPDS reduction) compared with 23.9% in the sham control arm [55].	Inclusion limited to moderate PPD, which limits generalizability [55]. No long-term follow-up data beyond 8 weeks; decentralized social media recruitment may limit generalizability [55].	Moderate evidence [55]
Digital Therapeutic Software for ADHD (n = 2)								
EndeavorRx	De Novo, DEN200026 (2020) [19]; 510(k), K231337 (2023) [20]	ADHD in children and adolescents aged 8–17 years with primarily inattentive or combined-type ADHD [19,20].	Game-based digital attention training methods through engaging game mechanics [19].	Initial: 4-week treatment, 25 min/day, 5 days/week. Pause: 4-week break. Repeat: optional second 4-week course if needed [19].	Systematic review and meta-analysis [50]. 14 RCTs pooled; n = 1183. Control group: Sham/active controls in RCTs.	Improvement in inattention and hyperactivity/impulsivity versus controls as assessed by parents and teachers; however, medication remained more effective [50].	Limited real-world transfer [51].	Moderate evidence [19,50,51]
Prismira	510(k), K243729 (2025) [21]	ADHD in adults aged 22–55 years with primarily inattentive or combined-type ADHD [21]; indicated to improve attention function, with the FDA caveat that patients may not display benefits in typical behavioral symptoms such as hyperactivity [21].	Prescription mobile software-as-medical-device; brief touchscreen games (1–5 min each) with visual and optional auditory feedback; integrated engagement system [21]	~15 min/day for 9 weeks [21]	GAMES Study—randomized, double-blind, parallel-group, multicenter, sham-controlled pivotal trial [21]. 560 enrolled; 456 ITT (active n = 224, sham n = 232). Control: sham digital program [21]	TOVA ACS at Day 63: LS-mean improvement 1.09 (active) vs. 0.30 (sham); LS-mean difference 0.78, 95% CI 0.15–1.41; *p* = 0.0149. CGI-I favored active treatment (*p* = 0.0087) [21] No serious adverse events; 2 treatment-related AEs (0.7%) [21]	No peer-reviewed full publication identified at synthesis (May 2026). Active-arm TOVA ACS improvement did not reach the established MCID of 1.4 points [21]. Most clinician- and patient-reported secondary endpoints (ADHD-RS, CBS, BRIEF-A, WFIRS-S) were not statistically significant [21]. Sponsor-conducted trial; FDA decision summary is the only currently available source [21].	Limited-to-moderate evidence [21].
Physiological Monitoring and Biofeedback / Wearable Devices (n = 3)								
Freespira	510(k), K131586 (2013) [22]; K180173 (2018) [23]	Panic Disorder; PTSD [56]	Capnometry Guided Respiratory Intervention: real-time feedback on respiratory rate and exhaled CO_2_ levels, aiming to normalize breathing patterns. Each session is 17 min; two sessions are recommended daily [56].	28 days [56]	Real-world effectiveness study [56]. n = 1569. Control group: None.	Panic disorder: 50.2% reduction in total PDSS scores; 65.3% achieving treatment response [56]. PTSD: 41.1% reduction in total PCL-5 scores; 72.4% treatment response rate [56].	Non-randomized design; no active control or respiratory placebo group; no extended follow-up to assess durability [56]. Breathing exercises alone may produce benefits independent of the biofeedback mechanism [56].	Limited evidence [56]
NightWare	De Novo, DEN200033 (2020) [24]	Nightmare disorder associated with PTSD [57]	AI-driven Apple Watch system monitors heart rate and motion during sleep; delivers calibrated vibrotactile stimulation when a nightmare-associated “Stress Index” threshold is crossed, interrupting nightmares without waking the patient [57].	30-day trial [57]	Randomized, double-masked, sham-controlled trial [57]. n = 65 veterans (30 active, 35 sham). Control group: Sham wearable device.	Both active and sham conditions demonstrated statistically significant within-person improvement across sleep quality, PTSD symptoms, and quality of life. No between-group difference reached statistical significance in the ITT analysis. Post hoc high-usage subgroup: significant improvement in perceived sleep quality with Active device [57].	Primary ITT outcome did not reach statistical significance; between-group effects only in a post hoc high-usage subgroup [57]. Small sample size (n = 65). No polysomnography or actigraphy collected. NightWare Likert (NWL) scale is not externally validated. Findings specific to VA veteran population with PTSD. No long-term follow-up beyond 30 days [57].	Limited evidence [57]
Prism	510(k) K222101 (2023) [25]	PTSD, adjunctive treatment [58]	Software-as-medical-device on a standard computer with supported EEG hardware (e.g., Nautilus PRO); operant-conditioning neurofeedback based on amygdala-derived electrical fingerprint (EFP) signals, with real-time visual and auditory feedback [58].	15 sessions × ≈25 min, twice weekly on non-consecutive days over 8 weeks [58]	Prospective, single-arm, multisite, multinational, open-label trial [58]. 79 enrolled (full analysis); 66 in effectiveness analysis set. Adults aged 22–65 with chronic PTSD [58]. Control group: None [58].	Primary endpoint met: 66.7% (44/66) of the effectiveness analysis set achieved a ≥6-point CAPS-5 reduction at 3-month follow-up (prespecified threshold ≥50%) [58]. Mean CAPS-5 reduction: 13.5 points (95% CI −16.4 to −10.0; *p* < 0.0001). CAPS-5 remission rate at 3 months: 31.8%. No device-related serious adverse events [58].	Single-arm, open-label design with no placebo, sham neurofeedback, or active comparator. Evaluated as an adjunct to the standard of care; not validated for acute PTSD [58]. US/OUS site response difference significant at 3 months but not at 8 weeks; effect absent in non-military trauma subgroup [58]. Multiple co-authors disclosed financial ties to the device developer/manufacturer (industry COI) [58].	Limited evidence [58]
Diagnostic Decision Support Devices for ASD (n = 2)								
CanvasDx	De Novo, DEN200069 (2021) [26]; 510(k), K243558 (2025) [27].	ASD diagnosis aid [26]	AI-based behavioral analysis using a gradient-boosted decision tree algorithm. Three inputs: caregiver questionnaire, two brief home videos of the child, and healthcare provider questionnaire. Outputs: ASD positive, ASD negative, or indeterminate [59].	Asynchronous; caregiver questionnaire (~5 min) and two brief home videos submitted via app; clinician questionnaire (~10 min) completed via web portal [60].	Pivotal diagnostic accuracy study [59]; real-world post-market analysis [60]. n = 425 [59]; n = 254 [60]. Control group: None; reference standard is specialist diagnosis.	In a study of 425 children (29% ASD): PPV 80.8%; NPV 98.3% [59]. Real-world post-market data: NPV 97.6%; PPV 92.4%; 63.0% of cases received determinate results [60].	Indeterminate outputs; the app abstains in a significant number of cases, especially when presentations are complex or comorbidities exist [59].	Moderate diagnostic-support evidence [59,60]
EarliPoint System	510(k), K213882 (2022) [28]; K230337 (2023) [29]; K243891 (2025) [30]; K253442 (2026) [31].	ASD diagnosis aid ages 16–30 months [61]	The system quantifies children’s visual focus on social stimuli by tracking eye movements while they watch brief video clips of naturalistic social scenes [61].	14 video scenes (mean ~54 s each; total ~12 min) [61]	Multisite prospective double-blind Phase 3 diagnostic accuracy study [61]. n = 475, aged 16–30 months. Control group: none; reference standard is specialist diagnosis.	Overall sensitivity 71.0%; specificity 80.7%. In the high-certainty subgroup of 335 children, sensitivity was 78.0%; specificity was 85.4% [61].	Not designed for general population screening [61]. Lead/senior authors are co-founders, equity holders, paid consultants, and patent holders for the device developer; two additional authors disclosed consultancy or institutional stock ties. Significant industry COI [61].	Moderate diagnostic-support evidence [61]
Computerized Attention Assessment Tools (n = 2)								
TOVA 9	510(k), K170082 (2017) [36]; K173915 (2018) [37]	ADHD diagnosis aid [37]	Continuous Performance Test (CPT) paradigm: presents visual or auditory stimuli; measures response times, variability in response times, omission errors (indicative of inattention), and commission errors (indicative of impulsivity) [37].	~21.6 min [37]	Systematic review and meta-analysis [62]. A total of 19 studies; up to 819 cases and 835 controls pooled. Control group: Varies across included studies.	Pooled sensitivity of 0.75 and specificity of 0.71 for the total/ADHD score across commercially available CPTs [62].	Limited diagnostic utility as a stand-alone tool [62]. Primary evidence [62] was not funded; 6/12 authors declared no biomedical COI; the remaining authors disclosed pharma and publisher royalty relationships, with no CPT developer specifically named.	Limited-to-moderate diagnostic-support evidence [37,62]
QbTest v3.5	510(k), K040894 (2004) [32]; K122149 (2012) [33]; K133382 (2014) [34]; K143468 (2016) [35]	ADHD diagnosis aid; treatment evaluation [33,34]	Combines continuous performance test (CPT) with infrared motion tracking technology to measure the three cardinal symptoms: inattention, hyperactivity, and impulsivity [63,64]	~15–20 min [63]	Systematic review and meta-analysis [64]; 15 studies. Control group: Varies across included studies.	Sensitivity 0.78; specificity 0.70, indicating a moderate discriminative capacity [64].	Limited diagnostic utility as a stand-alone tool [64]. Primary evidence [64] is not commercially funded, though some authors had prior professional ties to the QbTest developer or its regulatory review, representing a limited potential COI [64].	Limited-to-moderate diagnostic-support evidence [33,34,63,64]

**Narrative evidence appraisal:** Ratings were assigned pragmatically based on study design, comparator quality, endpoint interpretation, product-version relevance, independent replication, generalizability, and sponsor involvement. This was not a formal GRADE or risk-of-bias assessment. Moderate = at least one RCT, diagnostic accuracy study, or systematic review with important limitations. Limited-to-moderate = supportive evidence with major limitations, such as endpoint concerns, modest diagnostic utility, or limited replication. Limited = primarily uncontrolled, observational, post-market, post hoc, or nonrandomized evidence. Diagnostic-support products were appraised separately because they are aids to clinical assessment, not stand-alone diagnostic replacements. **Abbreviations:** BAT, behavioral activation therapy; CBT, cognitive behavioral therapy; CBT-I, CBT for insomnia; CPT, continuous performance test; DBT, dialectical behavior therapy; EFMT, emotional faces memory training; EPDS, Edinburgh Postnatal Depression Scale; GAD, generalized anxiety disorder; IPT, interpersonal therapy; ITT, intent-to-treat; IRT, imagery relief therapy; MADRS, Montgomery–Åsberg Depression Rating Scale; MDD, major depressive disorder; mITT, modified ITT; NPV, negative predictive value; OUD, opioid use disorder; PCL-5, PTSD Checklist for DSM-5; PDSS, Panic Disorder Severity Scale; PPD, postpartum depression; PPV, positive predictive value; PTSD, post-traumatic stress disorder; RCT, randomized controlled trial; SUD, substance use disorder; TAU, treatment as usual.

## 5. Discussion

### 5.1. Evidence Quality and Limitations

The current evidence base for FDA-authorized digital mental health products is heterogeneous and insufficient to support broad or definitive conclusions regarding clinical effectiveness. This variability limits direct comparisons across interventions, as products differ in intended use, mechanism of action, regulatory pathway, comparator design, outcome measures, and clinical role. For many products, the available evidence remains preliminary and heavily reliant on sponsor-generated studies or analyses involving authors with financial ties to developers. Many treatment studies employ waitlist controls [45,52], sleep hygiene education [48], sham devices [21,50,53,55,57], treatment-as-usual comparisons [48], or uncontrolled real-world designs [38,43,56] rather than gold-standard active comparators. This dependence on sponsor-associated evidence underscores the need for independent replication, transparent post-market evaluation, and cautious interpretation of efficacy claims.

A significant proportion of 510(k) clearances are based on substantial equivalence to predicate devices rather than direct evidence of clinical efficacy [9,65]. FDA guidance indicates that clinical data may be needed in specific circumstances, such as differences in intended use or technological characteristics [65]. The American Psychiatric Association (APA) [5] cautions that regulatory review status should not be interpreted as a comprehensive indicator of product quality or clinical effectiveness. Some clinical trials use prototypes [6] that differ from final marketed products, and the development of credible and inert digital placebos for sham controls remains a significant methodological challenge [6,66].

For diagnostic-support products, evidence should be interpreted in the context of their intended adjunctive role and their limited utility as stand-alone diagnostic replacements. A systematic review of continuous performance tests (CPTs) reported a pooled sensitivity of 0.75 and a specificity of 0.71, suggesting modest-to-moderate diagnostic utility as a stand-alone tool [62]. The American Academy of Pediatrics (AAP) guidelines state that neuropsychological testing generally does not improve diagnostic accuracy [67]. Post-market surveillance systems remain limited, particularly for software products that undergo frequent updates, which hinders monitoring of real-world safety and effectiveness [68,69]. Engagement and adherence remain problematic across the broader digital depression and anxiety app literature. While uptake in randomized controlled trials averages 92%, adherence, defined as completion of all modules, is only 59%, with considerable heterogeneity [70].

### 5.2. Predicate Propagation

The included products provide a concrete example of predicate propagation. EndeavorRx received a De Novo grant (DEN200026, 2020) [19] for ages 8–12 based on pediatric clinical evidence. A subsequent 510(k) clearance (K231337, 2023) [20] used the original EndeavorRx De Novo authorization as its predicate device and extended the indication to ages 8–17. Prismira (K243729, 2025) [21], a separate product from a different developer targeting adults aged 22–55, was subsequently cleared through the 510(k) pathway with EndeavorRx as the predicate. Therefore, Prismira’s adult clearance [21] is based on a predicate chain originating from an FDA decision supported by pediatric, rather than adult, efficacy data. This outcome reflects a structural feature of substantial-equivalence reasoning rather than a regulatory anomaly.

### 5.3. Commercial Discontinuity and Continuity of Care

Commercial discontinuity poses a structural risk to continuity of care that differs fundamentally from risks posed by drug or traditional device withdrawals. The bankruptcy of Pear Therapeutics [40] and subsequent acquisition or relaunch [41,42] of its digital products exemplify a unique implementation risk for digital therapeutics. Continuity of care may depend on software maintenance, ownership transitions, and commercial sustainability. This case demonstrates that the commercial viability of a digital therapeutic developer can affect ongoing patient access in ways not anticipated by conventional pharmacovigilance frameworks. Ensuring long-term access, data continuity, and platform support requires explicit consideration in clinical adoption decisions.

### 5.4. Privacy, Cybersecurity, and Data Governance

Privacy, cybersecurity, and data governance are essential considerations for clinical adoption. Digital mental health products may collect sensitive behavioral data, including electronic protected health information (ePHI) [5]. This may involve cloud storage, third-party analytics, or integration with clinical workflows. Clinicians and health systems should evaluate data-sharing practices, consent procedures, cybersecurity safeguards, and institutional privacy requirements prior to implementation.

### 5.5. Reimbursement and Clinical Integration

Access, coverage, and reimbursement are constrained by restrictive reimbursement policies and low clinician adoption, limiting access even after FDA marketing authorization. Although adoption is in its early stages, reimbursement pathways for digital therapeutics are emerging. The Centers for Medicare & Medicaid Services (CMS) has introduced new Medicare billing codes (HCPCS G0552-G0554) [71] for digital mental health treatment devices furnished incident to behavioral health services, and Cigna Healthcare has established commercial coverage criteria for qualifying prescription digital therapeutics [72,73]. These developments may indicate a trend toward broader adoption in the future.

The clinical integration of these products remains uncertain. At present, digital mental health products appear most suitable as low-intensity interventions for subclinical symptoms, as first-line treatments in stepped-care models, or as adjuncts to standard care [74]. Adoption remains limited; although providers report high interest, only 19% recommend digital products in practice, citing concerns about security, privacy, and workflow integration [5].

### 5.6. Regulatory Scope and Global Generalizability

This review examines the regulatory landscape of the United States Food and Drug Administration (FDA). In the United States, digital mental health products may reach the market through 510(k) clearance, De Novo classification, or, for higher-risk devices, premarket approval (PMA). These pathways differ in evidentiary requirements: 510(k) clearance is based on substantial equivalence to a legally marketed predicate device, De Novo classification provides a pathway for novel low- to moderate-risk devices, and PMA requires more extensive evidence of safety and effectiveness. De Novo devices may also serve as predicates for future 510(k) submissions, creating the possibility of predicate-chain effects across software product families.

In contrast, European market access occurs through CE marking under the European Union Medical Device Regulation (EU MDR) [75], which uses risk classification, conformity assessment procedures, clinical evaluation, and post-market surveillance obligations rather than the FDA’s 510(k), De Novo, and PMA pathway structure. These differences mean that FDA authorization does not ensure CE marking, reimbursement, clinical adoption, or equivalent evidentiary review in other jurisdictions. Therefore, the findings of this review should be interpreted as specific to FDA-authorized products in the United States. This review does not assess non-FDA-authorized digital mental health products, including products available through app stores, direct-to-consumer channels, or non-US regulatory pathways.

## 6. Conclusions

This narrative review identified 16 digital mental health products authorized by the FDA for psychiatric treatment or diagnostic support. The strength of the evidence base differs considerably among these products. Some are supported by randomized controlled trials or diagnostic accuracy studies, while others rely primarily on uncontrolled, observational, sponsor-generated, or post-market evidence. FDA marketing authorization should therefore be interpreted as a regulatory threshold rather than a guarantee of clinical effectiveness. Clinical implementation should consider the intended indication, the quality of supporting evidence, the comparator design, the relevance of the product version, safety and privacy concerns, and the feasibility of integration into clinical workflows.

## 7. Future Directions

Robust randomized controlled trials (RCTs) that utilize final marketed products, appropriate comparators, and clinically meaningful outcomes are necessary to generate high-quality evidence [6]. Clearer coding and coverage pathways are also essential to facilitate access [76]. Some reimbursement pathways have begun to emerge. For example, in November 2024, CMS finalized three HCPCS codes, G0552, G0553, and G0554, for digital mental health treatment devices furnished incident to professional behavioral health services [71], and in September 2025, Cigna Healthcare issued coverage criteria for qualifying prescription digital therapeutics [72,73]. Furthermore, clinical integration, provider training, and decision-support tools are needed to support adoption and advance the field.

## Figures and Tables

**Table 1:** FDA-authorized digital mental health products: results overview.

16 FDA-Authorized Digital Mental Health Products			
Product Category	Product	FDA-Authorized Use	Pathway
Treatment products (*n* = 12)	reSET	Substance use disorder (SUD), excluding patients on opioid replacement therapy, alcohol-only use, or primary opioid use.	De Novo classification
	reSET-O	Opioid use disorder (OUD) adjunct to outpatient treatment including buprenorphine.	510(k) clearance
	Somryst	Chronic insomnia.	510(k) clearance
	SleepioRx	Insomnia disorder.	510(k) clearance
	DaylightRx	Generalized anxiety disorder (GAD).	510(k) clearance
	Rejoyn	Major depressive disorder (MDD), adjunct to antidepressant medication; adults aged ≥ 22 years.	510(k) clearance
	MamaLift Plus	Mild to moderate postpartum depression (PPD); adults aged ≥ 22 years.	510(k) clearance
	EndeavorRx	ADHD; pediatric and adolescent (ages 8–17); inattentive or combined type.	De Novo classification + 510(k) clearance
	Prismira	ADHD; adults (ages 22–55); inattentive or combined type.	510(k) clearance
	Freespira	Panic disorder; PTSD.	510(k) clearance
	NightWare	Nightmare disorder associated with PTSD.	De Novo classification
	Prism	Posttraumatic stress disorder (PTSD).	510(k) clearance
Diagnostic-support products (*n* = 4)	CanvasDx	ASD diagnosis aid.	De Novo classification + 510(k) clearance
	EarliPoint System	ASD diagnosis aid (ages 16–30 months).	510(k) clearance
	TOVA 9	ADHD diagnosis aid.	510(k) clearance
	QbTest v3.5	ADHD diagnosis aid; treatment evaluation.	510(k) clearance

## Data Availability

No new data were created or analyzed in this study. Data sharing is not applicable to this article.
